# Ruminal volatile fatty acid absorption is affected by elevated ambient temperature

**DOI:** 10.1038/s41598-020-69915-x

**Published:** 2020-08-04

**Authors:** Andrea Bedford, Linda Beckett, Laura Harthan, Chong Wang, Ning Jiang, Hollie Schramm, Le Luo Guan, Kristy M. Daniels, Mark D. Hanigan, Robin R. White

**Affiliations:** 1 Department of Animal and Poultry Science, Virginia Tech, Blacksburg, VA 24061 USA; 2 Department of Dairy Science, Virginia Tech, Blacksburg, VA 24061 USA; 3 Large Animal Clinical Sciences, Virginia-Maryland College of Veterinary Medicine, Blacksburg, VA 24061 USA; 4 Faculty of Agricultural, Life & Environmental Sciences, University of Alberta, Edmonton, AB T6G 2P5 Canada; 5 Virginia Tech, 175 W. Campus Drive, 3300 Litton Reaves Hall, Blacksburg, VA 24073 USA

**Keywords:** Transcription, Metabolism

## Abstract

The objective of this study was to investigate the effect of short-term elevated ambient temperature on ruminal volatile fatty acid (VFA) dynamics and rumen epithelium gene expression associated with the transport and metabolism of VFA. Eight ruminally cannulated Holstein heifers (200 kg) were used in a factorial, repeated measures experiment with two treatments and two periods. During the first period, animals were provided with feed ad libitum and housed at 20 °C. During the second period, one group (HS) was housed at 30 °C and fed ad libitum. The other group (PF) was housed at 20 °C and pair-fed to match the intake of the HS group. During each period, animals were kept on treatment for 10 day, with sample collection on the final day. In the second period, indicators of heat stress were significantly different between PF and HS animals (*P* < 0.05). There was a thermal environment effect on butyrate production (*P* < 0.01) that was not associated with feed intake (*P* = 0.43). Butyrate absorption decreased in HS animals (*P* < 0.05) but increased in PF animals (*P* < 0.05) from period 1 to period 2. There was a feed intake effect on *BHD1* expression (*P* = 0.04) and a tendency for a thermal environment effect (*P* = 0.08), with expression increasing in both cases. Expression of *MCT4* was affected by feed intake (*P* = 0.003) as were all *NHE* genes (*NHE1*, *NHE2*, and *NHE3*; *P* < 0.05). These results indicate that with low feed intake and heat stress, there are shifts in rumen VFA dynamics and in the capacity of the rumen epithelium to absorb and transport VFA.

## Introduction

Climate change is a notable barrier to improving livestock sustainability because it may increase the incidence of heat stress (HS)^1^. Heat stress costs the U.S. livestock industries $1.7 billion annually^2^^,^ and leads to reduced growth rate^3^^,^ milk production^4^^,^ and reproductive efficiency^5,6^ in dairy cattle. Much of the loss in productivity is associated with reduced feed intake^7,8^ and increased maintenance energy^9^. Another key change that occurs during heat stress is that animals become insulin resistant and fail to mobilize body fat, favoring breakdown of muscle as means to obtain additional energy^10,11^. Although these shifts in post-absorptive metabolism have been well characterized in heat stressed cattle, changes in nutrient digestion and absorption have not been evaluated comprehensively. Multiple studies have shown changes in VFA concentrations related to rumen metabolism during heat stress^12–15^, which could be partially driven by a shift in intake. That being said, it is known that concentrations alone are not true representations of metabolism. In particular, absorbed volatile fatty acid (VFA) profiles may be implicated in the post-absorptive shifts in metabolism observed during heat stress because VFA have been shown to influence insulin sensitivity in monogastrics^16,17^. It is not unreasonable to think that a similar physiological response may also occur in ruminants.

We hypothesize that during exposure to elevated ambient temperature (HS treatment), shifts in the rumen microbiome and epithelium alter absorbed VFA profiles in comparison to pair-fed (PF control treatment) animals housed in thermoneutral conditions. Supporting objectives of the project were to: (A) measure changes in ruminal pH and VFA production and absorption during exposure to elevated ambient temperature; (B) assess how microbial gene expression for carbohydrate metabolism enzymes shifts during exposure to elevated ambient temperature; (C) characterize concurrent changes in gene expression in the rumen epithelium.

## Results

### Measures of thermal stress

Dry matter feed intake was significantly reduced in response to elevated ambient temperature (8.7 vs 6.9 kg/da in HS cows during period 1 and period 2, respectively; *P* < 0.01; Table 1). Period and treatment had a significant effect on rectal temperature, with HS heifers having a significantly higher temperature during period 2 compared to period 1 (38.6 vs 38.9 °C; *P* < 0.01; Table 1), and compared to PF cows in period 2 (38.5 vs 38.9 °C; *P* < 0.01; Table 1). There was a period × treatment interaction on respiration rate, with HS period 2 rates elevated over PF period 2 rates (45.4 vs 30.8 breaths/min; *P* < 0.01; Table 1), and on heart rate (*P* < 0.01). Because of these physiological shifts, period effects will hereafter be referred to as feed intake effects and interaction effects will reflect effects associated with elevated ambient temperature.

**Table 1:** Feed intake, vital signs, rumen pH and temperature associated with thermal stress or intake restriction.

Response	HS		PF		SE	P-values		
	P1	P2	P1	P2		Per	Trt	Per × Trt
Room temperature, °C	20.5^a^	29.0^b^	20.4^a^	20.0^a^	0.14	< 0.01	< 0.01	< 0.01
Dry matter intake, kg/day	8.7^b^	6.9^a^	9.2^b^	6.9^a^	0.5	< 0.01	0.69	0.44
Rectal temperature, °C	38.6^a^	38.9^b^	38.5^a^	38.5^a^	0.06	< 0.01	< 0.01	< 0.01
Respiration rate, breaths/min	40.4^b^	45.4^c^	34.2^ab^	30.8^a^	1.4	0.46	< 0.01	< 0.01
Heart rate, beats/min	75.6^b^	77.0^c^	68.4^a^	64.0^ab^	1.64	0.18	< 0.01	0.02
Median rumen temperature, °C	39.8^b^	39.2^a^	39.4^ab^	39.0^a^	0.11	< 0.01	0.08	0.06
Diurnal rumen temperature variation	3.9^a^	5.0^ab^	5.9^b^	4.3^ab^	0.52	0.67	0.31	< 0.01
Median rumen pH	6.3	6.2	6.3	6.2	0.13	0.08	0.92	0.35
Diurnal pH variation	6.8^a^	7.9^ab^	9.9^b^	6.3^ab^	0.74	0.10	0.41	< 0.01

*HS* Heat Stress*, PF* Pair-fed, *P1* period 1, *P2* period 2, *Per* period, *Trt* treatment, *Per* × *Trt* period by treatment interaction.

^a,b^Superscripts represent statistical differences among treatment groups a, b and periods.

### Rumen pH and temperature

There were no differences in median rumen pH across treatments (*P* > 0.05), but there was a trend for a feed intake effect (*P* = 0.08; Table 1). Feed intake did not have an effect on diurnal rumen temperature variation (*P* > 0.05), but there was an effect of elevated ambient temperature (*P* < 0.01; Table 1). There was a feed intake effect on median rumen temperature (*P* < 0.01; Table 1), with decreased temperatures in period 2, and a trend for an effect of elevated ambient temperature (*P* = 0.06), with decreased temperatures in PF cows (Table 1). Feed intake did not have an effect on diurnal pH variation (*P* > 0.05), however, there was an effect of thermal environment (*P* < 0.01; Table 1).

### Rumen VFA fluxes

Shifts in rumen VFA fluxes are shown in Table 2. VFA concentrations and molar proportions are shown in Supplemental Table 4. There was a trend for an increase in acetate production between periods (HS: 1.87 vs 2.13; PF: 2.67 vs 2.72; *P* = 0.06). Acetate absorption was increased in period 2 for both treatments (*P* < 0.01). Fluxes of both butyrate and propionate to acetate were affected by feed intake (*P* < 0.01), with both interconversions increasing. The flux of acetate to butyrate increased by reduced feed intake (*P* < 0.01), but the flux of acetate to propionate was unaffected (*P* = 0.30). There was a thermal environment effect on acetate washout (*P* < 0.01), though acetate washout was unaffected by feed intake (*P* = 0.89). There was a feed intake effect on propionate production (*P* < 0.01), with reduced feed intake leading to reduced production. Feed intake tended to affect propionate absorption (*P* = 0.06), with increased absorption when feed intake was reduced. There was a feed intake effect on the flux of butyrate to propionate (*P* < 0.01), with the flux increasing with decreased feed intake. There was a thermal environment effect on propionate washout (*P* < 0.05). Period also had an effect on propionate washout, increasing washout in HS cows, and decreasing it in PF cows (*P* = 0.02). The flux of propionate to butyrate was unaffected by either period or treatment (*P* = 0.33 and *P* = 0.92, respectively). There was an effect of elevated ambient temperature on butyrate production (*P* < 0.01), though it was unaffected by feed intake (*P* = 0.43). Butyrate absorption was affected by feed intake, with a decrease in animals exposed to elevated ambient temperature from period 1 to period 2, and an increase in pair fed animals from period 1 to period 2 (*P* = 0.01). There was also a thermal environment effect on butyrate absorption. Butyrate washout was affected by feed intake, with decreases in both HS and PF cows from period 1 to period 2 (*P* < 0.01). Total VFA production was unaffected by treatment. There was however a thermal environment effect on total VFA washout (*P* = 0.01), with a trend for a feed intake effect (*P* = 0.08).

**Table 2:** Shifts in VFA fluxes (mmol/h) associated with thermal stress and intake restriction.

Flux	HS			PF			P-values		
	P1	P2	SE	P1	P2	SE	Per	Trt	Per × Trt
Acetate production	1.87	2.13	0.26	2.67	2.72	0.30	0.06	0.13	0.18
Butyrate to acetate	1.07^a^	1.61^b^	0.32	0.53^a^	0.74^b^	0.36	< 0.01	0.19	0.20
Propionate to acetate	1.07^a^	1.76^b^	0.14	0.62^a^	1.41^b^	0.16	< 0.01	0.10	0.51
Acetate washout	0.883^a^	1.04^b^	0.16	1.14^b^	0.854^a^	0.18	0.33	0.89	< 0.01
Acetate absorption	0.648^a^	0.960^b^	0.19	0.820^a^	0.985^b^	0.22	< 0.01	0.74	0.30
Acetate to butyrate	1.18^a^	2.25^b^	0.54	1.11^a^	2.45^b^	0.62	< 0.01	0.93	0.53
Acetate to propionate	1.31	1.26	0.60	0.760	0.570	0.69	0.30	0.52	0.57
Propionate production	1.32^a^	1.08^b^	0.17	1.59^a^	1.19^b^	0.20	< 0.01	0.49	0.28
Butyrate to propionate	0.138^a^	1.30^b^	0.48	0.51^a^	1.88^b^	0.49	< 0.01	0.49	0.43
Acetate to propionate	1.31	1.26	0.60	0.76	0.570	0.69	0.30	0.53	0.57
Propionate washout	0.416^ab^	0.440^ab^	0.07	0.506^b^	0.348^a^	0.08	0.02	0.99	< 0.01
Propionate absorption	0.321^a^	0.387^b^	0.13	0.840^a^	0.869^b^	0.15	0.06	0.05	0.48
Propionate to butyrate	0.955	1.04	0.29	0.894	1.01	0.34	0.33	0.92	0.89
Propionate to acetate	1.07^a^	1.76^b^	0.14	0.623^a^	1.41^b^	0.16	< 0.01	0.10	0.51
Butyrate production	0.762^a^	0.866^b^	0.05	0.906^ab^	0.838^ab^	0.06	0.43	0.49	< 0.01
Propionate to butyrate	0.955	1.04	0.29	0.894	1.01	0.34	0.33	0.92	0.89
Acetate to butyrate	1.18^a^	2.25^b^	0.54	1.11^a^	2.45^b^	0.62	< 0.01	0.93	0.53
Butyrate washout	0.720^a^	0.690^b^	0.16	0.820^a^	0.580^b^	0.19	0.01	0.98	0.05
Butyrate absorption	0.965^a^	0.566^b^	0.21	1.05^ab^	1.11^ab^	0.24	0.01	0.36	< 0.01
Butyrate to propionate	0.138^a^	1.30^b^	0.48	0.51^a^	1.88^b^	0.49	< 0.01	0.49	0.43
Butyrate to acetate	1.07^a^	1.61^b^	0.32	0.53^a^	0.74^b^	0.36	< 0.01	0.19	0.20
Total VFA production^1^	3.95	4.08	0.35	5.17	4.75	0.40	0.37	0.12	0.10
Total VFA absorption^1^	1.93	1.91	0.18	2.71	2.96	0.21	0.24	0.02	0.16
Total VFA washout	2.02^a^	2.17^a^	0.39	2.46^a^	1.78^b^	0.44	0.08	0.96	0.01

*HS* Heat stress*, PF* Pair-fed, *P1* period 1, *P2* period 2, *Per* period, *Trt* treatment, *Per* × *Trt* period by treatment interaction.

^1^Acetate, butyrate, and propionate.

^a,b^Superscripts represent statistical differences between periods within animal groups.

### Microbial enzyme gene expression

The expression of genes with a significant thermal environment effect from liquid and solid rumen contents are presented in Tables 3 and 4. These tables also indicate probable nutrient targets for the genes. In liquid rumen content samples, thermal environment affected expression of 11 tested genes; expression of 24 genes was impacted by thermal environment in solid rumen content samples. When exposed to elevated ambient temperature, expression of genes related to digestion of cellulose and starch in the liquid rumen contents increased. In pair fed conditions, there was a decrease in the expression of genes related to the digestion of cellulose in the solid rumen contents.

**Table 3:** Gene expression in liquid rumen contents with significant interaction effects.

Gene product	Target/function	HS		PF		SE	P-value^^^
		P1	P2	P1	P2		
Alpha glucosidase	Multiple Targets	10.6^b^	9.74^ab^	9.12^a^	9.64^ab^	0.26	0.026
Amylase	Starch	10.0^a^	11.1^b^	10.9^b^	10.9^b^	0.12	0.001
Cellulase	Cellulose	5.47^a^	7.75^b^	7.27^ab^	7.53^b^	0.44	0.023
Endo-1-4-beta xylanase	Hemicellulose	8.07^b^	7.85^b^	6.96^ab^	6.09^a^	0.35	0.014
Pectate lyase	Pectin	7.36^b^	6.35^ab^	6.43^ab^	5.82^a^	0.27	0.021
Phosphorylase	Multiple Targets	6.43^ab^	7.67^b^	5.50^a^	7.62^b^	0.46	0.029
Pullulanase type 1	Multiple Targets	10.0^b^	9.36^ab^	8.56^a^	9.40^ab^	0.24	0.019
Alpha-N-aarabinofuranosidase	Hemicellulose	10.5^b^	9.74^ab^	10.0^ab^	9.46^a^	0.20	0.026
Cellulase	Cellulose	6.25^a^	8.69^b^	7.99^ab^	8.39^b^	0.47	0.025
Beta-xylosidase	Hemicellulose	8.53^b^	8.24^b^	6.81^a^	8.00^ab^	0.30	0.016
Glycogen synthase	Glucose	9.84^b^	8.27^a^	8.83^ab^	8.66^ab^	0.31	0.038

*HS* Heat Stress*, PF* Pair-fed, *P1* period 1, *P2* period 2.

^a,b^Superscripts represent statistical differences among treatment groups a, b and periods.

^^^P-values reflect the significance level for the interaction of period and treatment. Only those gene products with significant interaction effects are shown.

**Table 4:** Gene expression in solid rumen contents with significant interaction effects.

Gene product	Target	HS		PF		SE	P-value^^^
		P1	P2	P1	P2		
Acetylxylan esterase	Xylan	5.45^ab^	4.12^a^	6.32^b^	5.71^b^	0.33	0.009
Acetylxylan esterase	Xylan	7.68^b^	6.85^ab^	7.31^ab^	5.98^a^	0.35	0.044
Cellodextrin phosphorylase	Multiple targets	8.95^b^	8.21^ab^	8.44^ab^	7.19^a^	0.29	0.015
Cellulase	Cellulose	8.37^c^	7.49^b^	7.30^b^	6.18^a^	0.17	< 0.001
Cellulase	Cellulose	7.75^b^	7.08 ^ab^	7.33 ^ab^	6.04^a^	0.30	0.021
Cellulase	Cellulose	9.45^b^	8.36^ab^	8.58 ^ab^	7.91^a^	0.26	0.017
Cellulase	Cellulose	7.83^b^	6.76^b^	6.68^b^	5.23^a^	0.29	0.002
Chitinase	Chitin	4.83^b^	5.24^b^	5.28^b^	1.25^a^	0.65	0.006
Cyclomaltodextrinase		5.26^a^	7.16^b^	6.06^ab^	6.41^ab^	0.37	0.039
Endo-1-4-beta xylanase	Hemicellulose	8.51^b^	7.39^ab^	8.06^ab^	6.78^a^	0.35	0.035
Endo-1-4-beta xylanase	Hemicellulose	6.34^ab^	5.50^a^	7.23^b^	7.02^b^	0.32	0.019
Endo-1-4-beta xylanase	Hemicellulose	8.47^b^	6.98^ab^	7.88^ab^	6.35^a^	0.39	0.022
Endo-1-4-beta xylanase	Hemicellulose	9.37^b^	8.23^ab^	8.83^ab^	7.83^a^	0.26	0.014
Endo-1-4-beta xylanase	Hemicellulose	7.76^b^	6.50^a^	7.90^b^	7.40^ab^	0.22	0.008
Glucan-1-4-beta glycosidase	Multiple targets	6.34^b^	6.04^b^	4.66^b^	1.14^a^	0.59	0.001
Glucan-1-4-beta glycosidase	Multiple targets	6.27^ab^	7.30^b^	5.83^a^	6.69^ab^	0.28	0.031
Glycogen starch alpha glycan phosphorylase	Glycogen	8.72^c^	7.51^ab^	8.63^bc^	7.38^a^	0.25	0.009
Glycoside hydrolase	Multiple targets	9.45^b^	8.41^ab^	8.77^ab^	7.92^a^	0.24	0.012
Glucoside hydrolase	Multiple targets	7.87^b^	7.01^ab^	8.07^b^	5.57^a^	0.42	0.011
Glycosyltransferase	Multiple targets	6.17^a^	7.24^ab^	7.61^ab^	7.73^b^	0.34	0.043
Alpha glycanotransferase	Multiple targets	9.02^ab^	9.32^ab^	8.32^a^	9.36^b^	0.23	0.040
Feruloul esterase	Multiple targets	8.13^b^	7.11^a^	8.14^b^	7.48^ab^	0.18	0.010
Glucosidase	Multiple targets	7.15^ab^	6.46^a^	7.96^c^	7.51^bc^	0.16	0.001
1–2-Alpha L fucosidase	Fructose	6.16^b^	6.44^b^	4.90^a^	6.45^b^	0.36	0.046

*HS* Heat Stress*, PF* Pair-fed, *P1* period 1, *P2* period 2.

^a,b^Superscripts represent statistical differences among treatment groups a, b and periods.

^^^P-value associated with the interaction of period and treatment.

### Rumen epithelial gene expression

Gene expression data from the rumen epithelium are presented in Table 5. There was a tendency for a feed intake effect on *AACS, AKT1*, and *HMGCS2* expression, with levels decreasing, increasing, and increasing, respectively, in period 2 (*P* < 0.10). There was a thermal environment effect on the expression of *HSP70* (*P* < 0.05), and a tendency for a feed intake effect (*P* < 0.10), with lower expression with exposure to elevated ambient temperature and reduced intake. There was a feed intake effect on *BHD1* expression (*P* = 0.04), and a tendency for an effect of elevated ambient temperature (*P* = 0.08), with increased expression with reduced intake and exposure to high temperatures. There was a tendency for a thermal environment effect on *MCT1* expression (*P* = 0.08), with increased expression in period 2 PF animals compared to period 1 PF animals, but decreased expression in period 2 HS animals compared to period 1 HS animals. Expression of *MCT4* was affected by feed intake (*P* = 0.003) with increased levels with decreased intake. There was a significant thermal environment effect on expression of all NHE genes (*NHE1*, *NHE2*, and *NHE3*) (*P* < 0.05) with relative abundance decreasing in period 2 HS animals compared to period 1 HS animals and increasing in period 2 PF animals compared to period 1 PF animals. There were no treatment effects on expression of *HMGCL*, *CLDN1*, *GJA1*, or *MCT2* (*P* > 0.10).

**Table 5:** Gene expression in the rumen epithelium.

Gene	HS		PF		P-values		
	P1	P2	P1	P2	Trt	Per	Trt × Per
Acetoacetyl-CoA synthetase (*AACS*)	0.0002	0.0001	0.0004	0.0001	0.5053	0.0766	0.3310
3-Hydroxymethyl-3-methylglutaryl-CoA lyase (*HMGCL*)	1.14E−05	2.27E−05	4.86E−05	4.28E−05	0.2249	0.5127	0.3512
Heat shock protein 70 (*HSP70*)	0.0027^a^	0.0102^b^	0.0049^ab^	0.0045^ab^	0.7983	0.0521	0.0338
Serine-threonine protein kinase 1 (*AKT1*)	0.0016	0.0086	0.0014	0.0139	0.8205	0.0606	0.7324
3-Hydroxybutyrate dehydrogenase, type 1 (*BDH1*)	0.1434	0.1723	0.1058	1.0485	0.1543	0.0496	0.0815
3-Hydroxy-3-methylglutaryl-CoA synthase 2 (*HMGCS2*)	0.3562	0.6606	0.2919	3.0896	0.2936	0.1077	0.3112
Claudin 1 (*CLDN1*)	0.0383	0.0324	0.0242	0.0321	0.5281	0.8032	0.3491
Gap junction protein alpha 1 (*GJA1*)	0.0549	0.0436	0.0464	0.0502	0.9556	0.7936	0.5958
Monocarboxylic acid transporter 1 (*MCT1*)	0.0589	0.0275	0.0393	0.0522	0.6725	0.3728	0.0796
Monocarboxylic acid transporter 2 (*MCT2*)	1.90E−05	1.07E−05	8.85E−06	1.41E−05	0.5973	0.9020	0.2682
Monocarboxylic acid transporter 4 (*MCT4*)	8.32E−05	1.47E−04	3.54E−05	1.35E−04	0.0357	0.0036	0.1093
Sodium/hydrogen exchanger 1 (*NHE1*)	0.0009^a^	0.0005^b^	0.0004^b^	0.0007^ab^	0.6682	0.8368	0.0253
Sodium/hydrogen exchanger 2 (*NHE2*)	0.0305^a^	0.0133^b^	0.0127^b^	0.0208^ab^	0.3906	0.4734	0.0246
Sodium/hydrogen exchanger 3 (*NHE3*)	0.0044^a^	0.0013^b^	0.0011^b^	0.0026^ab^	0.5319	0.7016	0.0455

*HS* Heat Stress, *PF* Pair-fed, *P1* period 1, *P2* period 2, *Per* period, *Trt* treatment, *Per* × *Trt* period by treatment interaction.

^a,b^Superscripts represent statistical differences among treatment groups a, b and periods.

## Discussion

Given the impact heat stress has been shown to have on the performance of dairy cattle^18^, and how integral VFA dynamics are to the physiological status of the animal^19^, establishing a better understanding of the interaction between the two could greatly help with the nutritional management of these animals. In the present study, we used eight ruminally cannulated dairy heifers split into two groups to assess effects of thermal environment on VFA dynamics. While lactating dairy cows are more likely to experience substantial heat load during thermal stress and have differing metabolic demands, heifers were used in the current study as there were animal size constraints on our facilities. Nonetheless, the use of heifers provides valuable information on how the rumen responds during times of elevated ambient temperature.

As expected, exposure to elevated ambient temperature resulted in reduced feed intake, increased rectal temperature, and increased respiration rates. These measures are consistent with physiological responses commonly reported in heat stressed cattle^18^. Heart rate and respiration rate increased (*P* = 0.003 and *P* < 0.001, respectively) in HS during period 2, suggesting that the thermal treatment was sufficient to elicit a thermoregulatory response in animals. Along with the significant change in respiration and heart rates, body temperature increased in response to increased ambient temperature. It is common to observe rectal temperature changes in studies involving shifts in thermal environment; our results suggest animals were unable to employ thermoregulatory mechanisms successfully, including the redirection of blood flow and enhanced respiration, to maintain core body temperature within the normal range^18^. Our reported values for rectal temperature, while lower in absolute value, are in line with rectal temperature data from other experiments^20–22^.

When comparing the total VFA production, absorption, and washout data, there were no differences in total production, suggesting that any shifts in the production of individual VFAs were balanced out by another. Similarly, we saw no feed intake or thermal environment effects on individual VFA absorption rates. There was an effect of thermal environment on VFA washout, with period 2 pair fed animals having lower values compared to all other groups. The fact that a similar decrease was not observed from period 1 to period 2 in animals exposed to elevated ambient temperature suggests, but does not show, an ambient temperature effect on increasing washout that may otherwise be decreased by reduced feed intake.

Multiple shifts in the production of individual VFAs were observed related to feed intake and ambient temperature. The observed tendency for increased acetate production in period 2 compared to period 1, agrees with an increase of interconversion of butyrate to acetate, both directly and indirectly through propionate. Propionate production was decreased in both groups when feed intake was reduced. This was concurrent with an increased conversion of propionate to acetate with reduced feed intake. Propionate absorption was increased with reduced feed intake. It is likely that the reduced intake contributed to increased ruminal concentrations (Table S4) which promoted the increased absorption mediated by mass-action kinetics. During elevated ambient temperature, ruminal butyrate production increased, while a decrease was observed in pair fed animals. In both treatments there were increased interconversions of acetate to butyrate, along with increased interconversions of butyrate to acetate in period 2. These results indicate that while acetate and propionate dynamics appear linked with feed intake, thermal environment has a specific impact on butyrate dynamics.

The differences in VFA production observed with reduced feed intake are not readily explained by changes in rumen microbial gene expression observed here. When categorized by digestive substrate, reduced feed intake led to both increased and decreased expression of microbial genes encoding enzymes related to the digestion of simple sugars, cellulose, and hemicellulose. These results suggest that rumen VFA dynamics associated with reduced feed intake are intricate, and this is an area requiring more work to fully understand. Future work looking beyond enzymatic gene expression would help with understanding how heat stress affects rumen microbial function, including methanogenesis, and in turn, VFA dynamics.

On the other hand, we observed many interaction effects on rumen microbial gene expression that may shed some light on the differences in butyrate production observed with elevated ambient temperature. For instance, there were shifts in the expression of a number of genes for enzymes targeting cellulose, with moderate and significant decreases in the expression of a number of genes associated with hemicellulose and simple sugar metabolism. Each microbial species in the rumen has a substrate of preference and utilizes specific metabolic pathways^23^^,^ which gives rise to the profile of VFAs produced. Although the rumen microbiota was not characterized in the present study, it is possible that microbial population shifts occurred due to exposure to elevated ambient temperature, as reported by Uyeno et al.^24^. These shifts could have led to the changes in gene expression observed. The type of VFA produced is also dependent on the type of substrate fermented. The observed shifts in expression of microbial enzyme genes away from cellulose digestion in the solid rumen contents align with coefficients derived by Bannik et al.^25^ that would support a shift in VFA production towards butyrate.

The reported effects of heat stress on ruminal pH are varied. In the present study, there was a significant interaction effect on diurnal rumen pH variation, with variation increasing in animals exposed to elevated ambient temperature and decreasing in pair fed, thermoneutral counterparts. In grain-induced acidosis situations, rumen pH dynamics are thought to be partially driven by the behavior of the rumen epithelium because VFA absorption occurs partially through HCO_3_^-^ related mechanisms^26^. Divergent shifts in *MCT1* expression, as well as the expression of all sodium-hydrogen antiporters, support this hypothesis that the rumen pH dynamics are related to epithelial function. If the corresponding proteins follow the behavior of the *MCT1* gene expression, heat stressed animals could have reduced intracellular HCO_3_^-^ levels and reduced HCO_3_^-^ recycling into the rumen because influx from the blood would be lower. Further, the reduced *NHE* expression in the same animals would limit the buffering effect the epithelium would have on the rumen contents^27–29^. Rumen pH has been shown to have an effect on VFA absorption^30^, and the diurnal rumen pH variation observed here could have influenced the VFA absorption differences observed. Additionally, changes in diurnal rumen pH variation could be attributed to the role these transporters play in the absorption of VFAs from the rumen, and the effect ruminal VFA concentrations can have on pH^31^.

Differences in both acetate and butyrate absorption were observed with changes in intake and thermal treatment. There was an increase in acetate absorption with reduced feed intake, which could be a consequence of the increased expression of rumen epithelium transporters involved in VFA absorption observed. Elevated ambient temperature and pair fed animals differed in the washout of acetate, with washout increasing in animals exposed to the elevated temperature animals and decreasing in pair fed animals. This could be a result of increased water intake in the elevated thermal environment, which was not measured in this study, but is commonly observed^32–34^. Animals exposed to elevated ambient temperatures had reduced butyrate absorption. A large proportion of acetate is absorbed via diffusion^35^ while butyrate relies more on active transport. We propose that the reduced butyrate absorption in heat stressed animals is related to a backlog of VFAs in the rumen epithelium, resulting in reduced butyrate absorption due to a concentration gradient. In the rumen epithelium of heifers exposed to elevated ambient temperatures, we observed decreased expression of transporters responsible for the transport of VFA metabolites into the blood (*MCT1, MCT2, NHE1, NHE2*). The expression of the same transporters was increased in pair fed animals. The divergence of the expression of these transporters, as well as the increased expression of *HMGCL* and *BDH1*, which mediate the metabolism of acetoacyl-CoA to BHB within the epithelial cell, suggest a backlog of butyrate metabolite export in heat stressed animals. We propose that during exposure to elevated ambient temperature, increased acetate absorption in turn leads to increased metabolism of both acetate and butyrate to BHB, and decreased expression of transporters to move BHB out of the epithelial cell. These effects combine to produce a concentration gradient that reduces butyrate absorption from the rumen.

**Figure 1:**
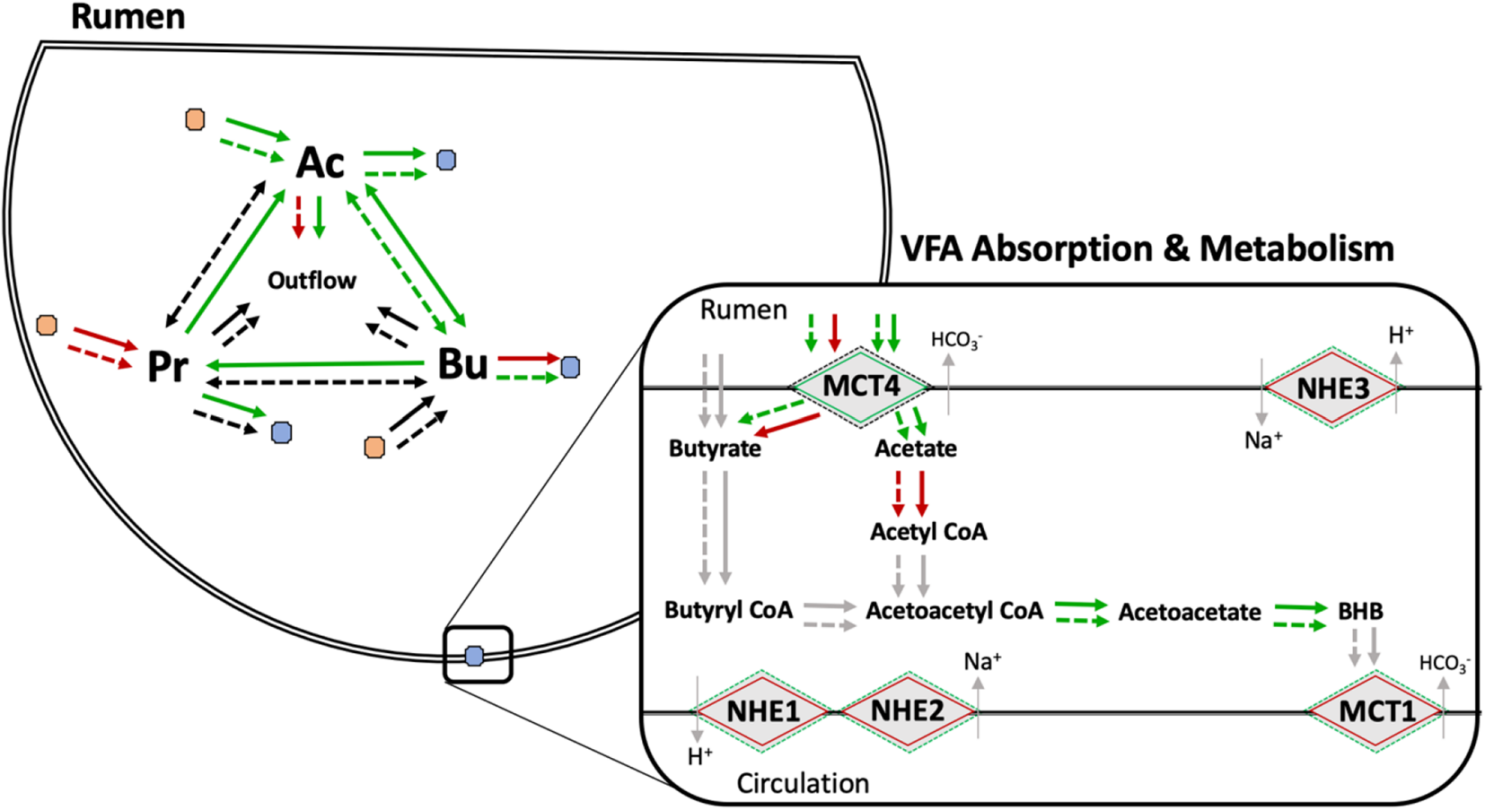
Summary of rumen dynamics influenced by elevated ambient temperature and feed intake. Rumen VFA fluxes including production from substrate (brown circle), absorption of VFA (blue circle), outflow with fluid, and interconversions, and intraepithelial VFA metabolism during heat stress (solid line) and feed restricted (dashed line) conditions. Significant increases are represented by green lines, significant decreases by red lines, no change by black lines, and unobserved by grey lines.

In conclusion, our results indicate that both thermal environment and feed intake can affect rumen VFA dynamics, including microbial gene expression, and gene expression in the rumen epithelium. In Fig. 1, we present a summary of the results discussed here. With low feed intake, the epithelium appears to increase capacity for VFA absorption and transport, perhaps in an effort to compensate and increase energy balance. However, during periods of elevated ambient temperature, it appears that butyrate production is increased, but the absorption and transport capacity to remove butyrate metabolites from the epithelium is decreased.

## Materials and methods

### Animals and experimental design

This experiment was approved by the Virginia Tech Institutional Animal Care and Use Committee (Protocol #15-177). All experiments were performed according to the guidelines and regulations set out by this governing body. Eight ruminally cannulated Holstein heifers (200 kg) were used in a factorial, repeated measures experiment with two treatments and two periods. Heifers were housed and fed individually in an indoor temperature-controlled facility for the duration of the experiment. During the first period, animals were provided feed ad libitum and exposed to constant 20 °C temperature. During the second period, one group (HS) was exposed to constant 30 °C temperature and were fed ad libitum. The other group (PF) was exposed to constant 20 °C but were pair-fed to match the intake of heifers in the HS group. During each period, animals were exposed to the thermal or intake treatment for 10 days, with the 3 days at the end of each period used for sample collection. A 7 days adaptation period was used because intake depression associated with elevated ambient temperature has been shown to stabilize (in the short term) after 5 to 7 days exposure. We attempted to assess what shifts in rumen fermentation kinetics occur independent from the depression in feed intake predicted to occur during exposure to elevated ambient temperatures. A snapshot of those changes during days 7 to 10 was deemed best for this assessment because allowing a longer adaptation period, as normally included in a nutrition trial, would neither reflect typical elevations in ambient temperature (which typically fluctuate over the short-term) nor the intake depression associated with short-term exposure to elevated ambient temperature.

### Feed intake and diet composition

Heifers in both groups were offered feed at 3% of BW (DM basis) daily during the first period, split into two meals (08:00 and 18:00 h) for days 1 through 6. Refused feed was collected prior to the morning feeding and weighed to estimate feed intake from the previous day. During days 7 through 10, animals were fed every 2 h to obtain a metabolic steady state beginning at 08:00 h, with the total feed offered per day equating to 3% of BW on a DM basis. During period 2, HS animals were offered feed at 3% of BW, split into two meals (08:00 and 18:00 h) for days 1 through 6. Refused feed was collected and weighed prior to each feeding to estimate ½ d intake. At each feeding, animals in the PF group were provided only the amount of feed consumed over the previous ½ d by the corresponding paired animal in the HS group; on days 7 through 10, this amount was divided accordingly. Diet and refusal samples were dried in a 55 °C forced-air oven (Thermo Scientific Heratherm Advanced Protocol Ovens Model 51028115; Fisher Scientific, Waltham, MA) for 48 h to analyze for DM content. Samples were ground with a Model 4 Wiley mill (A. H. Thomas Scientific, Swedesboro, NJ) to pass through a 1-mm screen. Ash was determined after heating for 8 h in a muffle furnace (500 °C). An Ankom200 fiber analyzer (Ankom Technology, Macedon, NY) with the addition of heat stable α-amylase and sodium sulfite was used to determine NDF. Feed ADF content was assessed using the Ankom200 fiber analyzer (Ankom Technology, Macedon, NY) according to manufacturer specifications. Crude protein was calculated as N × 6.25 after quantification of total N by combustion analysis (Vario El Cube CN analyzer, Elementar Americas Inc., Mount Laurel, NJ). Starch concentrations were determined using the acetate buffer method of Hall (2009) with α-amylase from *Bacillus licheniformis* (FAA, Ankom Technology, Macedon, NY) and amyloglucosidase from *Aspergillus niger* (E-AMGDF, Megazyme International, Wicklow, Ireland)^36^. Ration composition is presented in Table S1 and did not vary over the experimental period.

### Indicators of thermoregulation and heat stress

Throughout the experimental period, rectal temperature, respiration rate, and heart rate were measured twice daily at 06:00 and 18:00 h. Respiration rate and heart rate were measured using visual inspection and a stethoscope, respectively. Respiration rate and heart rate were observed to indicate thermoregulatory mechanisms being actively employed by the animal. Changes in rectal temperature were observed to represent the occurrence of acute heat stress.

### Ruminal pH and temperature

Ruminal pH and temperature were measured continuously over the duration of the experimental period using indwelling measurement boluses (Inovotec, Kirkwood, MO).

### Rumen sampling and infusions

On day 7 of each period, fluid sampling devices consisted of tygon tubing terminating in a pot scrubber, weighted with several steel nuts were installed in each animal and were threaded through holes in the cannula plug to maintain the anaerobic rumen environment. One pot scrubber was placed in the cranial portion of the rumen and one placed in the caudal portion. The ends of the tygon tubing were scored to allow a lure lok syringe to be screwed directly onto each tube. For each rumen fluid sample collected (detailed in the subsequent paragraph) a 60 mL syringe was then used to sample equal volumes of fluid from each sampling line. Samples were mixed in the syringe and the bulk sample was aliquoted into 2 glass vials and frozen until further analysis.

On days 8, 9 and 10 of each period, a rumen fluid sample was collected 15 m prior to the start of continuous infusions. This sample served as the zero-hour sample for isotope-based assessments. A pulse dose (400 mL) of a propylene glycol solution (PEG; 10% w/w) was then deposited directly into the rumen via the cannula over a 1-min timeframe (roughly 5 min prior to start of continuous infusion) to avoid overfilling and spillage. Rumen contents were then manually mixed to ensure dispersion of the infusate throughout the rumen and another rumen fluid sample was collected. This rumen sample served as the zero-hour sample for PEG-based assessments. Immediately after collecting this sample, continuous infusions were initiated. Continuous infusions were delivered intraruminally using Plum A + Infusion Systems (Abbott Laboratories, IL, USA). Isotope solutions were individually infused for 6 h at a rate of 100 mL/h on subsequent days (8, 9 and 10) starting at approximately 06:00 each day. Infusion order was randomized across animals. Isotopes were sourced from Cambridge Isotope Laboratories (Andover, MA) and included Na-2-^13^C-acetate (99% enrichment; solution concentration: 0.062%), Na-2-^13^C-propionate (99% enrichment; solution concentration: 0.062%), and Na-2-^13^C-butyrate (99% enrichment; solution concentration: 0.030%). These continuous infusions were pumped through tygon tubing threaded through the cannula plug and into a polyethylene bottle suspended in the rumen. The polyethylene bottle had holes bored in all sides and was filled with sponge material and weights to maintain its positioning in the rumen. This infusion distribution device was designed to partially sink in the rumen and diffuse infusate equally in all directions. The vessel was designed to allow mixing with the ruminal contents while ensuring that the infusate was not delivered near the rumen wall where the high osmotic potential and relatively low pH would cause irritation and tissue damage. After continuous infusions were initiated, rumen fluid samples (40 mL) were collected as described above every 1 h for 12 h (until approximately 18:00 h). After collection, samples were immediately frozen and maintained at − 20 °C until further analysis.

### Analytical methods for VFA concentration and isotope ratios

A total of 13 rumen fluid samples were collected for each infusion day for each animal, yielding a total of 39 samples per animal per period. The 13 samples collected on each day were pooled (100 μL per sample to create a 1.3 mL pooled sample) and used for determination of VFA concentrations. VFA concentrations were determined by isotope dilution using a constant externally added tracer 0.254 mM acetate-d3 (99 atom % D), 0.123 mM propionate-d5 (98 atrom % D), and 0.083 mM butyrate-d7 (98 atom % D) using a gas chromatograph (GC) coupled with an ion-trap mass spectrometer (MS) as previously described^37^. Following derivatization, samples were analyzed using a Thermo Electron Polaris Q mass spec (MS) in tandem with a Thermo Electron Focus Gas Chromatograph (GC) using XCalibur software (version 1.4). A Varian FactorFour capillary column VF-170 ms (30 m, 0.25 mm, 0.25 μm) was used. A total of 1 μL of sample was loaded with inlet temperature set to 225 °C on a split ratio of 80, running a constant flow of Helium carrier gas set to 1.2 mL/min. The GC was initiated at 75 °C, ramped at 5 °C/min to 135 °C, then at 40 °C/min to 225 °C. The MS was programmed to run in positive SIM mode collecting in three consecutive segments m/z pairs for acetate (43, 47), propionate (57, 59), and butyrate (71, 73) in that elution order. The processing method used to integrate the area under the curves for each m/z utilized the ICIS algorithm.

Individual samples (39 per animal period) were used for determination of isotope ratios. An isotope ratio mass spectrometer (IRMS; Delta V, Thermo Fisher Scientific, Waltham, MA) coupled to a gas chromatograph via a combustion oven (GC-comb-IRMS) was used to measure ^13^C enrichment of the CO_2_ arising from each VFA. VFA were introduced into the GC using a SPME method (SPME autosampler kit for Thermo Tri-Plus; SPME Fiber Assembly, 75um CAR/PDMS, 23ga, Autosampler (Supelco, P/N 57343-U); SPME Liner for TQ, 0.8 mm ID, Straight Through (Supleco, PN 2876601-U), Thermo Scientific). The SPME fiber was exposed to the headspace of each capped sample vial containing 600 ul of rumen fluid for 5 min after acidification with HCl and heating to 240 C for 1 min. The VFA were separated on a Zebron ZB-FFAP column, 30 m × 0.25 mm × 0.25 um (Phenomenex, P/N 7HG-G009-11) operated at 300^0^ C using Helium as a carrier gas at a flow rate of 1.5 mL/min. Data were expressed as isotope ratios. This approach has lower detection limits below 0.001% enrichment and all isotope infusion doses have been successfully detected in previous experiments.

### Dynamic isotope model fitting

Traditionally, isotope studies have relied on continuous infusions with production, clearance, and interconversion rates calculated from steady-state observations using a 3 pool model^38^ solved mathematically for 12 unknown rates^39,40^. An alternative non-steady state approach allowing for shorter infusion times was used by Nolan et al. In this work, we relied on the approach used by Nolan et al. Isotope ratios, VFA concentrations, and fluid volume and flow data were used to derive estimates of VFA production, absorption, and interconversion rates by fitting a dynamic mechanistic model of these fluxes. Production of acetate, propionate, and butyrate (mol/h) and 2-way interconversions of all VFA (%/h) were estimated by model fitting, fluid mediated exit of VFA (mol/h) was calculated based on VFA concentrations and measured fluid exit rates, and absorption of VFA was calculated by difference. The models were fit individually for each animal and infusion using the FME package of R (R Core Team). Residuals of all VFA concentration, isotope ratio, and fluid volume pools were evaluated for systematic biases, and when significant mean or slope biases were identified in these residuals, the models were re-fit using different parameter constraints until resulting residuals did not pattern systematically. For all final models, parameter values were used as starting values without constraints to check that the fitted model did not deviate when model constraints were removed. A summary of fit statistics for these models is presented in Table S2.

### Rumen microbial gene expression

On the final day of each period, approximately 2 h after the morning feeding, filtered rumen fluid samples (~ 50 mL) were obtained through a tygon sampling line of each heifer. Additionally, separate solid rumen content samples were collected by hand, through the open cannula, from 6 locations throughout the rumen fiber mat (~ 100 g obtained from the left and right regions of the cranial, medial, and caudal portions of the fiber mat). Rumen fluid and solid rumen content samples were individually snap frozen using liquid N. Once completely frozen, samples were crushed into small pellets, aliquoted, and stored at − 80 °C until further analysis. At a later date, ~ 30 mg of each sample was combined with 1 mL RNAzol-RT and 5 mg of 0.1 mm zircon beads and homogenized. After homogenization, samples were washed with 400 μL RNAse-free water, vortexed 15 s, and incubated at room temperature for 15 m. The supernatant was collected after centrifugation at 12,000×*g* for 15 min and mixed with equal volumes of ethanol. To eliminate genomic DNA contamination, isolated RNA was cleaned with a Zymo Research Clean & Concentrator kit. RNA quantity was evaluated by the Nanodrop method. RNA integrity were assessed using an Illumina Fragment Analyzer. Only samples with RNA integrity numbers greater than 7 were used for RNA sequencing; only one sample had insufficient RNA integrity for use. 100 ng of total RNA was used to construct an RNA library using the TruSeq RNA sample Prep v2 LS protocol (Illumina). RNA libraries were paired-end sequenced (2 × 150 bases) using an Illumina HiSeq2500 platform by Molecular Research LP (Shallowater, TX).

Microbial functional activity was analyzed as described in Li and Guan^41^. Briefly, the SortMeRNA program was used to increase putative mRNA as a percentage of total RNA by removal of rRNA from the total RNA sequences. Host mRNA was filtered from the sequence by aligning the reads to the bovine genome using TopHat. Filtered mRNA sequences were used for de novo assembly for each sample using MetaVelvet (kmer size of 31). Unique contigs were annotated using the UBLAST program of USEARCH against the CAZy database. A cutoff E value of < 1e−5, bit score of > 60 and sequence identity of > 30% were applied for annotation.

### Rumen epithelial gene expression

On the final day of each period, approximately 2 h after the morning feeding, rumen contents were partially evacuated through the rumen fistula of each heifer; the lateral, ventral portion of the rumen surface was brought to the fistula opening and surgical scissors were used to remove approximately 600 mg of rumen papillae. Samples were collected on the last day of each period to avoid any negative impacts of papillae biopsy on VFA absorption or microbial function. Papillae samples were immediately rinsed with ice cold NaCl solution (0.9%) to remove blood and large feed particles and stored in RNAlater (Thermo Fisher Scientific, Waltham, MA) at − 80 °C. Total RNA was later isolated from rumen papillae (~ 30 mg) with an RNeasy Plus kit (QIAGEN; Valencia, CA), which includes a genomic DNA removal step. Genes of interest and primer pairs used are presented in Table S2. Reverse transcription was conducted in an Arktik Thermal Cycler (Thermo Scientific; Waltham, MA). RNA purity and quantity were determined using a NanoDrop ND-1000 spectrophotometer (NanoDrop Technologies, Rockland, DE). Quantitative real-time reverse transcription PCR (qPCR) was performed using an Applied Biosystems 7500 Fast Real-Time PCR System (Thermo Fisher Scientific, Waltham, MA) with Fast SYBR Green Master Mix (Applied Biosystems, Thermo Fisher Scientific, Waltham, MA). Each reaction was performed in triplicate under the following conditions: one cycle at 95 °C for 20 s, 40 cycles of denaturation at 95 °C for 3 s and annealing at 60 °C for 30 s, and a subsequent melting curve (55–95 °C) with continuous fluorescent measurement. Relative mRNA levels were determined using the comparative Ct method, using ribosomal protein S15 (*RPS15*) as the internal reference gene. Each assay included a no-template control and a no-reverse-transcriptase control, with the no-template control receiving 1 μL of RNase/DNase free water instead of cDNA, whereas the no-reverse-transcriptase control received a 1 μL sample of the reverse transcription product to which no reverse transcriptase was added. The qPCR was repeated when the resulting intra-assay coefficient of variation was greater than 5%.

### Statistical analysis

Treatment efficacy was confirmed by evaluating room temperature, DMI and thermoregulatory response data using a linear mixed effect model which included effects for group, period, and the group by period interaction^42^. This same model structure was also used for assessing changes in VFA fluxes and rumen fluid volume and flow rate. Where multiple days of data were collected for a response variable, data were averaged over the experimental period (after 7 day adaptation to each period). Rumen pH and temperature data were evaluated by calculating the amount of time each animal spent under threshold pH or temperature values (thresholds = 4 to 7, by 0.1 for pH and 80 to 110 by 1 for temperature) and fitting a curve to the resultant data (numerous points, each reflecting a time under the threshold ranges previously noted). As described by Colman et al. the parameters of this curve reflect the median pH or temperature value and the diurnal variation in pH and temperature responses^43^. The resultant pH and temperature medians and diurnal variation estimates were assessed using the previously described mixed model. Rumen epithelial gene expression data were evaluated using the same model structure. Finally, responses in microbial gene expression were evaluated by estimating the differential expression of paired values. Differentially expressed genes associated with elevated ambient temperature versus pair-feeding (using only period 2 data) were assessed separately from differentially expressed genes associated with reduced feed intake (period 1 versus period 2 data). Differences were considered significant at P ≤ 0.05.

The datasets generated during and/or analysed during the current study are available from the corresponding author on reasonable request.

## Supplementary information

Supplementary information

## References

[1] Nardone A, Ronchi B, Lacetera N, Ranieri MS, Bernabucci U (2010). Effects of climate changes on animal production and sustainability of livestock systems. Livest. Sci..

[2] St-Pierre NR, Cobanov B, Schnitkey G (2003). Economic losses from heat stress by US livestock industries. J. Dairy Sci..

[3] Mader TL, Dahlquist JM, Hahn GL, Gaughan JB (1999). Shade and wind barrier effects on summertime feedlot cattle performance. J. Anim. Sci..

[4] Kadzere CT, Murphy MR, Silanikove N, Maltz E (2002). Heat stress in lactating dairy cows: a review. Livestock Prod. Sci..

[5] Hansen PJ (2001). Adverse impact of heat stress on embryo production: causes and strategies for mitigation. Theriogenology.

[6] Jordan ER (2003). Effects of heat stress on reproduction. J. Dairy Sci..

[7] Mader TL, Davis MS (2004). Effect of management strategies on reducing heat stress of feedlot cattle: Feed and water intake1. J. Anim. Sci..

[8] Spiers DE, Spain JN, Sampson JD, Rhoads RP (2004). Use of physiological parameters to predict milk yield and feed intake in heat-stressed dairy cows. J. Therm. Biol..

[9] Birkelo CP, Johnson DE, Phetteplace HP (1991). Maintenance requirements of beef cattle as affected by season on different planes of nutrition. J. Anim. Sci..

[10] Rhoads ML (2009). Effects of heat stress and plane of nutrition on lactating Holstein cows: I. Production, metabolism, and aspects of circulating somatotropin. J. Dairy Sci..

[11] Baumgard LH, Rhoads RP (2013). Effects of heat stress on postabsorptive metabolism and energetics. Annu. Rev. Anim. Biosci..

[12] Bianca W (1965). Reviews of the progress of dairy science. J. Dairy Res..

[13] Kelley RO, Martz FA, Johnson HD (1966). Effect of environmental temperature on ruminal volatile fatty acid levels with controlled feed intake. J. Dairy Sci..

[14] Martz FA, Mishra M, Campbell JR, Daniels LB, Hilderbrand E (1971). Relation of ambient temperature and time postfeeding on ruminal, arterial and venous volatile fatty acids, and lactic acid in Holstein steers. J. Dairy Sci..

[15] Schneider PL, Beede DK, Wilcox CJ (1988). Nycterohemeral patterns of acid-base status, mineral concentrations and digestive function of lactating cows in natural or chamber heat stress environments. J. Anim. Sci..

[16] Gao Z (2009). Butyrate improves insulin sensitivity and increases energy expenditure in mice. Diabetes.

[17] Lin HV (2012). Butyrate and propionate protect against diet-induced obesity and regulate gut hormones via free fatty acid receptor 3-independent mechanisms. PLoS ONE.

[18] West JW (2003). Effects of heat-stress on production in dairy cattle. J. Dairy Sci..

[19] Huntington GB (1990). Energy metabolism in the digestive tract and liver of cattle: influence of physiological state and nutrition. Reprod. Nutr. Dév..

[20] Carvalho FA, Lammoglia MA, Simoes MJ, Randel RD (1995). Breed affects thermoregulation and epithelial morphology in imported and native cattle subjected to heat stress. J. Anim. Sci..

[21] Gaughan JB, Mader TL, Holt SM, Josey MJ, Rowan KJ (1999). Heat tolerance of Boran and Tuli crossbred steers. J. Anim. Sci..

[22] Srikandakumar A, Johnson EH (2004). Effect of heat stress on milk production, rectal temperature, respiratory rate and blood chemistry in Holstein, Jersey and Australian Milking Zebu cows. Trop. Anim. Health Prod..

[23] Russell JB, Baldwin RL (1978). Substrate preferences in rumen bacteria: evidence of catabolite regulatory mechanisms. Appl. Environ. Microbiol..

[24] Uyeno Y (2010). An rRNA-based analysis for evaluating the effect of heat stress on the rumen microbial composition of Holstein heifers. Anaerobe.

[25] Bannink A (2006). Estimation of the stoichiometry of volatile fatty acid production in the rumen of lactating cows. J. Theor. Biol..

[26] Penner GB, Steele MA, Aschenbach JR, McBride BW (2011). Ruminant nutrition symposium: molecular adaptation of ruminal epithelia to highly fermentable diets. J. Anim. Sci..

[27] Sehested J, Diernaes L, Moller PD, Skadhauge E (1996). Transport of sodium across the isolated bovine rumen epithelium: interaction with short-chain fatty acids, chloride and bicarbonate. Exp. Physiol..

[28] Müller F, Aschenbach JR, Gäbel G (2000). Role of Na+/H+ exchange and HCO3- transport in pHi recovery from intracellular acid load in cultured epithelial cells of sheep rumen. J. Comp. Physiol. B.

[29] Gäbel G, Aschenbach JR, Müller F (2002). Transfer of energy substrates across the ruminal epithelium: implications and limitations. Anim. Health Res. Rev..

[30] Dijkstra J, Boer H, Van Bruchem J, Bruining M, Tamminga S (1993). Absorption of volatile fatty acids from the rumen of lactating dairy cows as influenced by volatile fatty acid concentration, pH and rumen liquid volume. Br. J. Nutr..

[31] Dijkstra J (2012). Ruminal pH regulation and nutritional consequences of low pH. Anim. Feed Sci. Technol..

[32] Rungruang S (2014). A dose-response evaluation of rumen-protected niacin in thermoneutral or heat-stressed lactating Holstein cows. J. Dairy Sci..

[33] Lamp O (2015). Metabolic heat stress adaption in transition cows: differences in macronutrient oxidation between late-gestating and early-lactating German holstein dairy cows. PLoS ONE.

[34] Yadav B (2016). Effect of simulated heat stress on digestibility, methane emission and metabolic adaptability in crossbred cattle. Asian-Austral. J. Anim. Sci..

[35] Storm AC, Kristensen NB, Hanigan MD (2012). A model of ruminal volatile fatty acid absorption kinetics and rumen epithelial blood flow in lactating Holstein cows. J. Dairy Sci..

[36] Hall MB (2009). Determination of dietary starch in animal feeds and pet food by an enzymatic-colorimetric method: collaborative study. J. AOAC Int..

[37] Erwin ES, Marco GJ, Emery EM (1961). Volatile fatty acid analyses of blood and rumen fluid by gas chromatography. J. Dairy Sci..

[38] Nolan JV, Leng RA, Dobos RC, Boston RC (2014). The production of acetate, propionate and butyrate in the rumen of sheep: fitting models to 14C- or 13C-labelled tracer data to determine synthesis rates and interconversions. Anim. Prod. Sci..

[39] Esdale WJ, Broderick GA, Satter LD (1968). Measurement of ruminal volatile fatty acid production from alfalfa hay or corn silage rations using a continuous infusion isotope dilution technique. J. Dairy Sci..

[40] Dijkstra J, Forbes JM, France J (2005). Quantitative Aspects of Ruminant Digestion and Metabolism.

[41] Li F, Guan LL (2017). Metatranscriptomic profiling reveals linkages between the active rumen microbiome and feed efficiency in beef cattle. Appl. Environ. Microbiol..

[42] O’Brien MD, Rhoads RP, Sanders SR, Duff GC, Baumgard LH (2010). Metabolic adaptations to heat stress in growing cattle. Domest. Anim. Endocrinol..

[43] Colman E, Tas BM, Waegeman W, De Baets B, Fievez V (2012). The logistic curve as a tool to describe the daily ruminal pH pattern and its link with milk fatty acids. J. Dairy Sci..

