# Supplementary material for: Ruminal volatile fatty acid absorption is affected by elevated ambient temperature
Source: Sci Rep. 2020 Aug 4;10:13092. doi: 10.1038/s41598-020-69915-x (PMC7403581; doi:10.1038/s41598-020-69915-x)
Supplement: Supplementary file 1 — Supplementary information [file 41598_2020_69915_MOESM1_ESM.docx]

***Title*:** Ruminal Volatile Fatty Acid Absorption is Affected by Elevated Ambient Temperature

***Authors*:** Andrea Bedford^1^, Linda Beckett^1^, Laura Harthan^2^, Chong Wang^2^, Ning Jiang^2^, Hollie Schramm^3^, Le Luo Guan^4^, Kristy M. Daniels^2^, Mark D. Hanigan^2^, and Robin R. White^1*^

***Affiliations*:**

^1^Department of Animal and Poultry Science, Virginia Tech, Blacksburg, VA, USA 24061

^2^Department of Dairy Science, Virginia Tech, Blacksburg, VA, USA 24061

^3^Large Animal Clinical Sciences, Virginia-Maryland College of Veterinary Medicine, Blacksburg VA 24061

^4^Faculty of Agricultural, Life & Environmental Sciences, University of Alberta, Edmonton, Alberta, Canada T6G 2P5

*Correspondence to: Robin White; 175 W. Campus Drive, 3300 Litton Reaves Hall, Blacksburg VA, 24073; rrwhite@vt.edu

**Supplemental Table 1.**  Diet Composition

| **Ingredient** | **% DM** |
| --- | --- |
| Corn Silage (37% DM) | 48.3 |
| Alfalfa Hay (90% DM) | 25.8 |
| Wheat Middlings | 7.66 |
| Ground Corn | 3.73 |
| Distillers Dried Grains | 1.48 |
| Gluten Feed | 5.10 |
| Soybean Meal | 4.79 |
| Soybean Hulls | 2.36 |
| Limestone | 0.36 |
| Salt | 0.28 |
| Trace Mineral Premix^1^ | 0.016 |
| Vitamin E | 0.026 |
| Vitamin A, D3, E Premix^2^ | 0.021 |
| Rumensin | 0.016 |
| 0.06% Selenium Premix^3^ | 0.052 |
| Dry Matter, % | 64.5 |
| Crude Protein, % DM | 15.1 |
| Neutral Detergent Fiber, % DM | 38.4 |
| Acid Detergent Fiber, % DM | 23.4 |
| Crude Fat, % DM | 3.07 |
| Starch, % DM | 20.6 |
| Ash, % DM | 6.84 |

^1^12% Ca (ground limestone, monocalcium phosphate, dicalcium phosphate), 4.0% P (monocalcium phosphate, dicalcium phosphate), 20% Na (salt, sodium selenite), 10% Mg (magnesium oxide, magnesium sulfate), 0.32% S (potassium sulfate, magnesium sulfate, zinc sulfate, ferrous sulfate, copper sulfate), 1.0% K (potassium chloride, potassium sulfate), 65 mg/kg I (ferrous sulfate, ethylenediamine dihydroiodide), 1,000 mg/kg Cu (copper sulfate), 30 mg/kg Co (cobalt carbonate), 100 mg/kg Se (sodium selenite), 5,000 mg/kg Zn (zinc oxide), and 1,500 mg/kg Mn (manganous oxide)

^2^451,000 IU/kg Vitamin A (vitamin A supplement), 123,000 IU/kg Vitamin D (vitamin D supplement), and 495 IU/kg Vitamin E (vitamin E supplement)

^3^0.06% Se (sodium selenite)

**Supplemental Table 2**. Concordance Correlation Coefficients for Models Describing Enrichment of Acetate, Propionate, and Butyrate Pools for Each Animal During Each Period

| **Animal** | **Period** | **Acetate** | **Propionate** | **Butyrate** |
| --- | --- | --- | --- | --- |
| 27 | 1 | 0.78 | 0.80 | 0.83 |
| 27 | 2 | 0.83 | 0.82 | 0.87 |
| 28 | 1 | 0.78 | 0.79 | 0.73 |
| 28 | 2 | 0.85 | 0.81 | 0.71 |
| 29 | 1 | 0.73 | 0.77 | 0.83 |
| 29 | 2 | 0.74 | 0.78 | 0.84 |
| 30 | 1 | 0.74 | 0.78 | 0.84 |
| 30 | 2 | 0.74 | 0.78 | 0.84 |
| 31 | 1 | 0.74 | 0.81 | 0.73 |
| 31 | 2 | 0.75 | 0.81 | 0.73 |
| 32 | 1 | 0.74 | 0.83 | 0.74 |
| 32 | 2 | 0.73 | 0.79 | 0.77 |
| 34 | 1 | 0.73 | 0.75 | 0.76 |
| 34 | 2 | 0.75 | 0.76 | 0.67 |
| 39 | 1 | 0.77 | 0.78 | 0.70 |
| 39 | 2 | 0.87 | 0.79 | 0.74 |

**Supplemental Table 3**. Primers

| **Primer** | **Sequence** | **Accession Number** | **Amplicon size (bp)** |
| --- | --- | --- | --- |
| Heat shock protein 70 (HSP70) | Fwd: AGCTGGAGCAGGTGTGTAAC  Rev: AGCTTGCATAGCTGATGGCT | U09861.1 | 239 |
| Serine-threonine protein kinase 1 (AKT1) | Fwd: CTGCACAAGCGAGGTGAGTA  Rev: GAAGTTGTTGAGGGGCGACT | NM_173986.2 | 132 |
| Sodium/hydrogen exchanger 1 (NHE1) | Fwd: GTCCCACACGACCATCAAGT  Rev: AGGGTGCTGATGACAAACGT | NM_174833.2 | 132 |
| Sodium/hydrogen exchanger 2 (NHE2) | Fwd: CGAGCAGCTCTACATCCTGG  Rev: ATGCCAGCAAACACGTCAAC | XM_604493.6 | 129 |
| Sodium/hydrogen exchanger 3 (NHE3) | Fwd: CCTCATGAGAAGGTCGGCTC  Rev: GAACGGATGAAAGCCAGGGA | NM_001192154.1 | 129 |
| Monocarboxylic acid transporter 1 (MCT1) | Fwd: GTCATTGGAGGTCTTGGGCT  Rev: GGTAGAGAGGAACACAGGGC | NM_001037319.1 | 129 |
| Monocarboxylic acid transporter 2 (MCT2) | Fwd: TGGTCTCGGCCTCTTACAGT  Rev: GCCATTCGCTACAGGTCGTT | NM_001076336.2 | 146 |
| Monocarboxylic acid transporter 4 (MCT4) | Fwd: GTGACACAGCCTGGATCTCC  Rev: AGAAGGACGCAGACACCATG | NM_001109980.1 | 150 |
| Gap junction protein alpha 1 (GJA1) | Fwd: ATGAGCAGTCTGCCTTTCGT  Rev: AGCCAGGTACAGGAGTGTGG | NM_174068.2 | 143 |
| Claudin 1 (CLDN1) | Fwd: CAGTGCAAAGTCTTCGACTCC  Rev: GTCGTCTTCCATGCACTTCA | BT021861.1 | 147 |
| Ribosomal protein S15 (RPS15) | Fwd: CAAGGCCAAGAAAGATGCGC  Rev: TCAGGCTTGATTTCCACCTGG | NM_001024541.2 | 147 |
| 3-Hydroxybutyrate dehydrogenase, type 1 (BDH1) | Fwd: CCCACCACCAGTCTGAGCAT  Rev: CCCACTACTCTGCACCCCAA | NM_001034600.1 | 101 |
| Acetoacetyl-CoA synthetase (AACS) | Fwd: ACCTGCGTGTGTGCCATTAC  Rev: TTAAGGCTGCAAGCTGGTTGA | NM_001163929.1 | 103 |
| 3-Hydroxymethyl-3-methylglutaryl-CoA lyase (HMGCL) | Fwd: ATTGTGGAAGTTGGTCCTCGA  Rev: GGACCCAGTGGCTCACAGTT | NM_001075132.1 | 105 |
| 3-Hydroxy-3-methylglutaryl-CoA synthase 2 (HMGCS2) | Fwd: TTACGGGCCCTGGACAAAT  Rev: CACATCATCGAGAGTGAAAGG | NM_001045883.1 | 100 |

**Supplementary Table 4. VFA Concentrations and Molar proportions**

| **Type** | **VFA** | **HS^1^** | | **PF^2^** | | **SE** | **P-Values** | | |
| --- | --- | --- | --- | --- | --- | --- | --- | --- | --- |
|  |  | **P1^#^** | **P2^$^** | **P1** | **P2** |  | **Per^%^** | **Trt^^^** | **Per x Trt^&^** |
| Concentration | Acetate | 46.8 | 52.8 | 39.5 | 51.0 | 9.26 | 0.034 | 0.569 | 0.494 |
|  | Propionate | 14.8 | 14.9 | 11.6 | 14.0 | 1.86 | 0.337 | 0.403 | 0.395 |
|  | Butyrate | 17.9 | 16.9 | 13.9 | 16.9 | 3.31 | 0.641 | 0.649 | 0.350 |
|  | Valerate | 1.21 | 1.47 | 0.983 | 1.58 | 0.256 | 0.013 | 0.868 | 0.313 |
|  | Isovalerate | 0.697 | 0.827 | 0.682 | 0.916 | 0.125 | 0.035 | 0.820 | 0.537 |
|  | Isobutyrate | 1.33 | 1.63 | 1.25 | 1.74 | 0.214 | 0.004 | 0.958 | 0.464 |
|  | Total VFA | 82.7 | 67.9 | 86.1 | 88.6 | 11.4 | 0.120 | 0.571 | 0.420 |
| Molar Ratio | Acetate | 58.1 | 59.9 | 58.5 | 60.5 | 1.26 | 0.034 | 0.722 | 0.887 |
|  | Propionate | 18.4 | 17.0 | 17.0 | 16.0 | 0.64 | 0.004 | 0.192 | 0.628 |
|  | Butyrate | 19.6 | 18.7 | 20.2 | 18.6 | 1.62 | 0.202 | 0.915 | 0.687 |
|  | Valerate | 1.40 | 1.62 | 1.43 | 1.71 | 0.10 | <0.001 | 0.661 | 0.701 |
|  | Isovalerate | 0.836 | 0.915 | 0.992 | 1.05 | 0.05 | 0.056 | 0.071 | 0.808 |
|  | Isobutyrate | 1.71 | 1.83 | 1.84 | 2.04 | 0.09 | 0.010 | 0.186 | 0.487 |

^1^ Heat Stress; ^2^ Pair-fed;  ^#^ P1: period; ^$^ P2: period 2; ^%^ Per: period; ^^^ Trt: treatment; ^&^ Per x Trt: period by treatment interaction
